# Confirming the Validity of the School-Refusal Assessment Scale—Revised in a Sample of Children With Attention-Deficit/Hyperactivity Disorder

**DOI:** 10.3389/fpsyg.2022.849303

**Published:** 2022-03-07

**Authors:** Stian Orm, Cathrine Orm, Mette I. Mebostad, Anders Dechsling, Anders Nordahl-Hansen

**Affiliations:** ^1^Division of Mental Health Care, Innlandet Hospital Trust, Brumunddal, Norway; ^2^Department of Education, ICT and Learning, Østfold University College, Halden, Norway

**Keywords:** school refusal, functional assessment, psychometric properties, confirmatory factor analysis, attention-deficit/hyperactivity disorder (ADHD)

## Abstract

Children with developmental disorders, such as attention-deficit/hyperactivity disorder (ADHD), are at high risk of school-refusal behavior (SRB) compared with their peers. One of the most used scales to assess SRB is the school refusal behavior scale – revised (SRAS-R). The SRAS-R has demonstrated good psychometric properties when used with the general population of children, but, recently, its validity has been questioned when used with children with developmental disorders. We tested the psychometric properties of the SRAS-R parental reports in 96 children with ADHD (M_age_ = 12.4; SD = 1.7, 61.5% boys). Results showed good model fit and internal consistency for the original four-factor structure. Three of the factors were strongly correlated, suggesting that SRB among children with ADHD is caused by several factors.

## Introduction

Attention-deficit/hyperactivity disorder (ADHD) is one of the most common developmental disorders, affecting around 5.9% of children and youth (Faraone et al., 2021). Children with ADHD present with academic and social difficulties at school, have a high school absence, and many display school-refusal behaviors (SRB; Martin, 2014; Fleming et al., 2017; Orm et al., 2020). However, currently, it is scarce with research on SRB among children with ADHD (Orm et al., 2020). For research, as well as clinical practice, it is important with valid scales to assess SRB among children with ADHD. Scales validated with neurotypical children are not necessarily valid among children with developmental disorders (Adams et al., 2021). Therefore, there is a need for studies examining the validity of SRB scales among children with developmental disorders such as ADHD.

A seminal paper by Kearney and Silverman (1993) divided SRB into four different behavioral functions: (1) avoidance of emotionally aversive (i.e., anxiety-provoking) situations, (2) escape from socially evaluative situations, (3) pursuit of attention from parents and significant others, and (4) pursuit of reinforcement outside of school. The school-refusal assessment scale (Kearney and Silverman, 1993), which has later been revised (SRAS-R; Kearney, 2002, 2006), was introduced with these four factors/subscales. The SRAS-R is commonly used for the assessment of SRB both in research and in clinical practice and has been translated into several languages (Gonzálvez et al., 2016, 2018; Walter et al., 2018; Filippello et al., 2020). The SRAS-R has been applied in clinical samples of juvenile adolescents and adolescents referred for treatment of SRB in the United States and Germany (Kearney, 2002, 2006; Walter et al., 2018), as well as general population samples of children and adolescents in Spain and Chile (Gonzálvez et al., 2016, 2018). Some studies have focused on the child report (Gonzálvez et al., 2016, 2018), whereas others have included both child and parent reports (Kearney, 2002, 2006; Walter et al., 2018). For the parent report, the factor structure has been confirmed in confirmatory factor analysis (CFA) and adequate internal consistency (α ≥ 0.70) for the four subscales, and convergent validity with measures of mental health has been demonstrated in clinical samples of adolescents (Kearney, 2006; Walter et al., 2018). However, Kearney (2006) found that a good fit on all CFA indices was contingent on removing items 18, 20, and 24. In the original exploratory factor analysis of the SRAS-R (Kearney, 2002), items 20 and 24 were found to load on a different factor from the other items related to Function 4 (seeking attractive activities outside of school), and Kearney (2006) suggests that this may be because of more complex wording, making these two items more difficult to interpret.

The four factors described in the SRAS-R describe two different functions of reinforcement – namely positive and negative reinforcements (Kearney, 2006). Positive reinforcement (3 and 4 above) describes maintenance or increase of behavior contingent on producing stimuli, while negative reinforcement (1 and 2) describes the same but is contingent on the removal of aversive stimuli. It might be difficult to assess whether SRB has the function of either one of 1 or 2, or 3 or 4. Therefore, it has been suggested that a two-factor solution (positive vs. negative reinforcement) may better capture the reasons for SRB (Kearney, 2006). In a neurotypical sample, however, the four-factor solution was superior to the two-factor solution (Kearney, 2006).

The SRAS-R may be particularly useful for research and work with high risk SRB populations, such as children with developmental disorders like ADHD. However, scales validated for use with neurotypical children cannot automatically be assumed to be equally valid when applied with children with developmental disorders. A recent study has found that the SRAS-R parent report had unsatisfactory psychometric properties in a sample of children (mean age = 12) with autism spectrum disorder (Adams et al., 2021); the original four-factor structure showed poor model fit in CFA. The lack of model fit for the four-factor solution could suggest that another factor structure (e.g., the simpler two-factor solution discussed above) may be more suitable for children with autism spectrum disorder and other developmental disorders.

Given the findings of Adams et al. (2021), an investigation of the factor structure and reliability of the SRAS-R when used with children with other types of developmental disorders (e.g., ADHD) is warranted. The current study aimed to examine the validity and reliability of the four-factor solution of the SRAS-R in a sample of children with ADHD and to compare the four-factor solution to a simpler and more parsimonious two-factor model.

## Method

### Procedure and Participants

Parents of 96 children with ADHD were recruited through social media groups for parents of children with ADHD, and the authors’ professional and social networks. The parents were asked to complete an anonymous, online questionnaire, including demographic information and the SRAS-R. The study was prospectively reviewed and approved by the Norwegian Center for Research Data. The children with ADHD were between 10 and 15 years of age (M = 12.4; SD = 1.74), and the majority were boys (61.5%). The majority of the responding parents were mothers (*n* = 92) as opposed to fathers (*n* = 4) and had university or college education of 1 year or more (73%).

### Measure

The parents completed the parent version of the SRAS-R (Kearney, 2002), which comprises 24 items rated on a Likert scale from 0 to 6 (never to always). Previous factor analyses have suggested that the SRAS-R can be divided into four different factors based on the function of the SRB (Kearney, 2002, 2006). These four factors are: (1) avoidance of aversive situations at school, (2) avoidance of social situations, (3) seeking attention from significant others, and (4) seeking tangible reinforcements outside of school. These subscales have demonstrated good internal consistency (α = 0.78–0.88) and test-retest reliability (*r* = 0.61–0.78) in previous studies (Kearney, 2002, 2006). In this study, we used the official Norwegian translation (Holden and Sållman, 2010). The Norwegian translation comprises the same 24 items as the original version. To date, no study has examined the psychometric properties of Norwegian translation.

### Data Analyses

Analyses were performed in JASP (JASP Team, 2020). Confirmatory factor analysis was performed with *diagonally weighted least square* (DWLS) estimation since the scale is ordinal (DiStefano and Morgan, 2014). The following fit indices were used as indicators of good model fit: non-significant chi-square, *comparative fit index* (CFI; ≥ 0.96), *root mean square error approximation* (RMSEA; ≤ 0.08), and standardized *root mean square residual* (SRMR; ≤ 0.09) (Mueller and Hancock, 2001; Alhija, 2010). Based on the previous literature (Kearney, 2002, 2006) suggesting that Items 20 and 24 poorly relate to the other items on Factor 4, we removed Items 20 and 24 before conducting a second CFA of the four-factor solution. Correlations between the four factors were examined with Pearson’s correlation coefficient (*r*), and scale reliability was assessed with McDonald’s omega (ω) and Cronbach’s alpha (α) as measures of internal consistency (values ≥ 0.70 indicate acceptable reliability). Pearson’s *r* of 0.10, 0.30, and.50, respectively, were interpreted as small, medium, and large correlations.

## Results

CFA testing the two-factor model showed poor model fit [CFI = 0.97, RMSEA = 0.070, *p* = 0.023, χ^2^ (251) = 367.433, *p* < 0.001, SRMR = 0.114, see Table 1]. Initial CFA of the four-factor model with all items included showed partial fit. Good fit was indicated based on CFI (= 0.98) and RMSEA (= 0.050, *p* = 0.502); chi-square test [χ^2^ (246) = 303.501, *p* = 0.007] and SRMR (= 0.104) indicated poor fit. In the CFA of the four-factor solution without Items 20 and 24, all fit indices indicated a good to excellent model fit [CFI = 1, RMSEA < 0.0001, *p* = 1, χ^2^ (203) = 177.207, *p* = 0.904, SRMR = 0.087].

**TABLE 1:** Results from confirmatory factor analyses and reliability analyses of the school-refusal assessment scale – revised (SRAS-R).

	Two-factor solution^1^			Four-factor solution			Adapted four-factor solution		
Factor (subscale)	Estimate	95% CI		Estimate	95% CI		Estimate	95% CI	
Item		Lower	Upper		Lower	Upper		Lower	Upper
**Factor 1 (avoidance)**									
Item 1	1.35	1.22	1.47	1.40	1.27	1.54	1.40	1.27	1.54
Item 5	1.16	1.03	1.28	1.21	1.08	1.34	1.22	1.09	1.36
Item 9	1.23	1.11	1.35	1.28	1.15	1.41	1.27	1.15	1.40
Item 13	1.42	1.29	1.55	1.48	1.33	1.62	1.47	1.33	1.61
Item 17	1.73	1.57	1.88	1.80	1.64	1.97	1.80	1.64	1.96
Item 21	1.41	1.28	1.54	1.48	1.34	1.62	1.47	1.33	1.61
Cronbachs alpha (α)	−	−	−	0.93	0.89	0.95	0.93	0.89	0.95
Mcdonalds omega (ω)	−	−	−	0.93	0.90	0.95	0.93	0.90	0.95
**Factor 2 (social avoidance)**									
Item 2	0.85	0.73	0.96	0.96	0.83	1.08	0.97	0.84	1.10
Item 6	0.80	0.70	0.91	0.91	0.79	1.03	0.90	0.77	1.03
Item 10	0.69	0.59	0.80	0.81	0.68	0.93	0.79	0.67	0.91
Item 14	1.32	1.19	1.45	1.46	1.31	1.62	1.52	1.36	1.68
Item 18	1.35	1.19	1.51	1.56	1.37	1.74	1.45	1.26	1.64
Item 22	1.40	1.27	1.54	1.56	1.40	1.71	1.58	1.42	1.74
Cronbachs alpha (α)	0.92	0.89	0.94	0.85	0.78	0.89	0.85	0.78	0.89
Mcdonalds omega (ω)	0.92	0.88	0.94	0.84	0.76	0.89	0.84	0.76	0.89
**Factor 3 (seeking attention)**									
Item 3	1.49	1.35	1.64	1.52	1.37	1.67	1.54	1.39	1.69
Item 7	1.20	1.08	1.33	1.22	1.09	1.35	1.22	1.09	1.35
Item 11	1.64	1.49	1.79	1.66	1.51	1.82	1.65	1.50	1.81
Item 15	1.21	1.06	1.36	1.23	1.08	1.38	1.23	1.08	1.39
Item 19	1.15	1.01	1.29	1.16	1.02	1.30	1.14	1.00	1.28
Item 23	1.67	1.51	1.84	1.70	1.53	1.87	1.69	1.52	1.87
Cronbachs alpha (α)				0.90	0.86	0.93	0.90	0.86	0.93
Mcdonalds omega (ω)				0.89	0.85	0.93	0.89	0.85	0.93
**Factor 4 (tangible reinforcements)**									
Item 4	0.01	−0.10	0.11	0.06	−0.10	0.23	1.04	0.72	1.35
Item 8	−0.16	−0.26	−0.05	0.29	0.13	0.46	0.86	0.60	1.12
Item 12	−0.03	−0.14	0.07	0.12	−0.05	0.28	1.42	1.00	1.84
Item 16	0.34	0.24	0.44	−0.58	−0.76	−0.40	0.16	−0.07	0.39
Item 20	0.85	0.69	1.01	−1.58	−1.99	−1.16	−	−	−
Item 24	0.30	0.19	0.42	−0.51	−0.70	−0.32	−	−	−
Cronbachs alpha (α)	0.80	0.73	0.85	0.53	0.32	0.67	0.68	0.54	0.77
Mcdonalds omega (ω)	0.81	0.74	0.86	0.54	0.40	0.65	0.74	0.66	0.82

*^1^Factors 1 and 2 and factors 3 and 4, respectively, were collapsed into a negative reinforcement and a positive reinforcement factor.*

*^2^Estimates for the collapsed factor of negative reinforcement comprising factors 1 and 2.*

*^3^Estimates for the collapsed factor of positive reinforcement comprising factors 3 and 4.*

*CI = confidence interval.*

The first factor (avoidance of aversive situations at school) correlated strong and significantly positively [*r* (95) = 0.64, *p* < 0.001] with the second factor (avoidance of social situations), meaning that children scoring high on one of the two functions tend to score high on the other function. The same applied to the first and the third factors (seeking attention from significant others), which also correlated strong and significantly positively [*r* (95) = 0.68, *p* < 0.001], and the second and the third factors [*r* (95) = 0.53, *p* < 0.001]. The fourth factor (seeking reinforcement outside of school) did not correlate significantly with any of the other factors [*r* (95) ≤ 0.07, *p* ≥ 0.482]. Analyses of internal consistency showed good to excellent reliability for the first, second, and third factors, and acceptable reliability for the fourth factor (see Table 1).

## Discussion

This study enhances our knowledge about the psychometric properties of the SRAS-R and SRB among children with ADHD in at least three ways: (1) by demonstrating the suitability of the SRAS-R for children with ADHD in terms of both confirming the original factor structure and showing adequate reliability, (2) providing the first data on the psychometric properties of the Norwegian translation of the SRAS-R, suggesting the psychometric properties of the original version can be extended to the Norwegian context (at least in the case of children with ADHD), and (3) by comparing the four-factor structure to a simpler and theoretically justified two-factor structure, suggesting that the more complex four-factor structure is, in fact, a better representation of SRB in children with ADHD.

Comparing our results with previous studies, it seems like the psychometric properties of the SRAS-R, when used with children with ADHD, are more similar to a neurotypical sample (Kearney, 2006) than an autism spectrum disorder sample (Adams et al., 2021). Thus, the SRAS-R seems to be adequate for assessing the reasons for SRB in children with ADHD but not children with an autism spectrum disorder. In common with the study of Kearney (2006), we found that the four-factor solution was superior to the simpler and more parsimonious two-factor structure. This finding suggests that the reasons for SRB among children with ADHD are more nuanced than just positive and/or negative reinforcement. It is necessary to also take into consideration the type of stimuli (e.g., social, non-social). However, three of the four factors (#1, 2, 3) showed large correlations with each other, suggesting that there will often be several reasons for SRB among children with ADHD, related to both positive and negative reinforcements. At the same time, the finding that the fourth factor (seeking reinforcement outside of school) was completely unrelated to the other factors is interesting. This suggests that children with ADHD who score high on the fourth factor display SRB only to achieve reinforcements outside of school, perhaps because they are bored at school, but, to a lesser extent because they experience emotional discomfort when attending school (Filippello et al., 2018). These children may require a different interventional approach compared to those experiencing emotional discomfort at school (i.e., negative reinforcement Factors #1 and 2). Whereas components of anxiety treatment often will be necessary when intervening with children displaying SRB under negative reinforcement, approaches emphasizing monitoring and reinforcement of attendance will be critical for children scoring high on the fourth factor (Kearney, 2008).

Our study provides the first evidence of the practical utility of the SRAS-R when assessing SRB among children with ADHD and the psychometric properties of the Norwegian translation. This is important because teachers, educational psychological services, and child and adolescent mental health centers need psychometrical sound measures to assess SRB among these children. This is because children with ADHD are at higher risk of problematic school absence, school suspension, and SRB, but, at the same time, little is known about the reasons, and the reasons may differ considerably between individuals (Kearney, 2008; Fleming et al., 2017; Orm et al., 2020; Sultan et al., 2021). The SRAS-R represents a relatively brief and clinically useful assessment scale for teachers and clinicians to get more information about the reasons and motivating factors for SRB in each case (Kearney, 2002, 2006).

Children with ADHD generally present with elevated levels of mental health problems compared with neurotypical children (e.g., Orm et al., 2021). As such, assessments of SRB in children with ADHD should be supplemented with mental health assessments, as this can provide additional information about the reasons for SRB. For example, internalizing behavior problems, such as anxiety and/or depressive symptoms, may relate more closely to the first three factors compared with the fourth factor (Kearney and Albano, 2004; Gonzálvez et al., 2021). The relationship between mental health problems and SRB may also be more specific, such as the second factor (social avoidance) relating to social anxiety disorder and the third factor (seeking attention from significant others) relating to separation anxiety disorder (Kearney and Albano, 2004). Conversely, the fourth factor may be more closely tied to externalizing behavior problems (Egger et al., 2003; Kearney and Albano, 2004). Thus, treatment of mental health problems in children with ADHD may prevent or contribute to the treatment of SRB. However, future studies are needed to assess the relations between mental health problems and SRB in children with ADHD, as we cannot automatically assume that the relationships identified in studies of neurotypical children also apply to children with ADHD.

Although the SRAS-R, in general, seems like a practical scale to get insights into the reasons for SRB and plan support and interventions accordingly, more knowledge is needed about which inter- and intrapersonal variables affect SRB in children with ADHD and how these variables relate to the different factors of SRB outlined in the SRAS-R. In addition to mental health problems, studies examining the impact of executive functioning and social skills would be particularly interesting. This is because deficits in both executive functioning and social skills are hallmarks of ADHD (Storebø et al., 2019; Fossum et al., 2021), and are related to SRB in children with autism spectrum disorder (Munkhaugen et al., 2019). Moreover, social and emotional difficulties have been found to associate with higher school absences among children with ADHD (Classi et al., 2012). Whereas mental health problems, executive functioning, and social skills are relevant intrapersonal variables to include in future studies, researchers should not overlook interpersonal variables. Variables relating to the school climate, such as position and popularity in the peer group, stigmatization and bullying, and the teacher-student relationship, are relevant to include in future studies. A recent study found that bullying was related to SRB in children with ADHD but having 1:1 aid decreased this risk (McClemont et al., 2021). Future studies examining the impact of the aforementioned variables on the SRAS-R factors can provide us with important information for targeting educational support and interventions.

### Limitations

The most notable limitation of the current study is the relatively modest sample size for CFA, making replications in larger samples warranted. However, our sample size was close to that of previous CFA studies of the SRAS-R (e.g., Kearney, 2006; Adams et al., 2021; *N* = 115 and 121). Furthermore, when interpreting the results, emphasis was placed on fit indices relatively robust to small sample sizes (i.e., CFI, RMSEA, SRMR) instead of the chi-square statistic, which may fail to reject an unfitting model with a small sample. We also used DWLS estimation rather than the more commonly used maximum likelihood (ML) due to its suitability for ordinal scales but also its robustness when used with small samples (Brown, 2015). Still, replications are needed, and studies comparing the factor structure of the SRAS-R across children with ADHD and autism spectrum disorder would be particularly interesting to see how these two related developmental disorders differ in their reasons for SRB. Another limitation of this study is the lack of more information related to the participants’ socioeconomic status. We included parents’ educational levels as a proxy of socioeconomic status as educational level correlates with other socioeconomic factors such as income and employment (Baker, 2014), but more information on socioeconomic status would have helped to contextualize our sample even further.

## Data Availability Statement

The raw data supporting the conclusions of this article will be made available by the authors, without undue reservation.

## Ethics Statement

Ethical review and approval was not required for the study on human participants in accordance with the local legislation and institutional requirements. The patients/participants provided their written informed consent to participate in this study.

## Author Contributions

MM, CO, SO, and AN-H contributed to conception and design of the study. MM, CO, and AN-H were in charge of data collection. MM, CO, SO, AD, and AN-H planned the statistical analyses. MM, CO, and SO performed the statistical analysis. SO wrote the first draft of the manuscript. MM, CO, AD, and AN-H wrote sections of the manuscript. All authors contributed to manuscript revision, read, and approved the submitted version.

## Conflict of Interest

The authors declare that the research was conducted in the absence of any commercial or financial relationships that could be construed as a potential conflict of interest.

## Publisher’s Note

All claims expressed in this article are solely those of the authors and do not necessarily represent those of their affiliated organizations, or those of the publisher, the editors and the reviewers. Any product that may be evaluated in this article, or claim that may be made by its manufacturer, is not guaranteed or endorsed by the publisher.
